# Experimental and Modeling Study on the Aging Behavior of Silicone Rubber Foam: A Simplified Ogden Approach with a Single Time-Varying Parameter

**DOI:** 10.3390/polym18111344

**Published:** 2026-05-28

**Authors:** Haiyan Li, Gui Huang, Ming Guo, Fei Wu, Biao Li, Xin Xie

**Affiliations:** Naval University of Engineering, Wuhan 430033, China; 1712021038@nue.edu.cn (H.L.); 1920191070@nue.edu.cn (F.W.); 1708051051@nue.edu.cn (B.L.); 1708051053@nue.edu.cn (X.X.)

**Keywords:** silicone rubber foam, aging constitutive model, Ogden model, accelerated thermal aging, compression set, stress relaxation, single time-varying parameter, hyperelasticity

## Abstract

Silicone rubber foam is widely used in multi-field engineering protection due to its excellent cushioning and thermal insulation properties. However, its performance degradation caused by long-term service aging seriously affects equipment reliability. Establishing a constitutive model that can accurately characterize the mechanical response during aging is crucial for studying performance degradation and finite element simulation. Traditional multi-parameter aging constitutive models suffer from problems such as easy convergence to local optimal solutions and poor physical interpretability of parameters. To address these issues, this study systematically characterizes the evolution laws of the stress–strain response, compression set, and stress relaxation of silicone rubber foam over an aging period of 0–768 h through accelerated thermal aging and uniaxial compression tests and proposes a second-order Ogden aging constitutive model with a single time-varying parameter. This model fixes α_1_, α_2_, and μ_2_ as constants and only sets μ_1_ as the time-varying parameter, reducing the number of parameters to be fitted from four to one. The coefficient of determination (R^2^) of the full-cycle stress–strain curve fitting is ≥0.9966. Meanwhile, a quantitative physical correlation between μ_1_ and macroscopic aging performance indicators is established, enabling the direct prediction of the mechanical response of aged materials using measurable macroscopic indicators. This work provides an efficient and reliable modeling method for the aging performance evaluation and structural simulation of silicone rubber foam.

## 1. Introduction

Silicone rubber foam is widely used in engineering systems as impact-absorbing cushions, liners, and thermal insulation materials due to its excellent thermal stability, high energy absorption, and favorable damping properties [1,2]. In such applications, foam components are often exposed to various environmental stresses—including temperature, mechanical stress, humidity, radiation, and chemical media—over service periods ranging from several years to decades. The long-term effects of these environmental stresses inevitably lead to physical and chemical changes in the rubber foam, such as the formation of oxidation products and new crosslinked structures, backbone scission, and collapse of the porous structure. These changes result in a gradual decline in mechanical performance, thereby shortening the service life of components.

The aging behavior of silicone rubber foam can be evaluated through performance indicators such as stress–strain curves, compression set, stress relaxation, elongation at break, and tensile strength [3,4,5,6,7,8]. Among these, the stress–strain response most directly reflects the aging state of the material and has therefore attracted considerable attention. Many studies have attempted to incorporate environmental stress and time parameters into constitutive models to describe the aging effects in silicone rubber foam [9,10,11]. Currently, aging constitutive models for rubber are predominantly phenomenological in nature [12,13]. For example, Lou et al. [14] developed a thermal aging constitutive model based on the Ogden model by establishing relationships between the model parameters and temperature as well as aging time. Similarly, Liu et al. [13] established an aging constitutive model for ethylene-propylene-diene monomer (EPDM) rubber by correlating the parameters of a first-order Mooney–Rivlin model with pre-compression and aging time.

It is generally believed that higher-order models offer a more accurate description of the stress–strain response. However, excessively high model orders can lead to multi-parameter fitting issues, which are prone to generating multiple local optima, undermining model reproducibility [15,16], and making it difficult to accurately reveal the evolution of parameters with aging time. In constructing an aging constitutive model for 3D printed polymer foam, Maiti et al. found that, while maintaining fitting accuracy, keeping most model parameters constant and allowing only a few to vary with time can effectively reduce the number of local optima and better establish the relationship between parameters and aging time [17].

In this study, high-temperature accelerated aging tests were conducted on silicone rubber foam under compression to obtain data on stress–strain curves, compression set, and stress relaxation. Based on the experimental data, the degradation trajectories of compression set and stress relaxation over aging time were obtained. Using the Ogden model, the constitutive model was reasonably simplified by limiting the number of time-dependent parameters. Based on the physical meaning of the parameters, functional relationships were established between these parameters and compression set as well as stress relaxation, thereby predicting the variation trend of the parameters over time. This work provides a new approach and methodology for developing aging constitutive models for silicone rubber foam.

## 2. Second-Order Ogden Constitutive Model

The second-order Ogden hyperelastic model is widely employed to describe the mechanical behavior of silicone rubber foam, owing to its excellent fitting accuracy [14,17,18,19]. Its strain energy function is defined as

(1) $$U\left(\lambda_{1},\lambda_{2},\lambda_{3}\right)=\overset{2}{\underset{i=1}{\sum }}\frac{2\mu_{i}}{\alpha_{i}^{2}}\left\{\lambda_{1}^{\alpha_{i}}+\lambda_{2}^{\alpha_{i}}+\lambda_{3}^{\alpha_{i}}-3+\frac{1}{\beta_{i}}\left[{\left(\lambda_{1}\lambda_{2}\lambda_{3}\right)}^{-\alpha_{i}\beta_{i}}-1\right]\right\}$$

where $\lambda_{1}$, $\lambda_{2}$, and $\lambda_{3}$ are the principal stretches in three orthogonal directions; $\alpha_{i}$ represents the hardening/softening exponents that characterize the nonlinearity of the stress–strain response; and $\mu_{i}$ and $\beta_{i}$ (*i* = 1,2) are model parameters, which are related to the material parameters (initial shear modulus $\mu_{0}$ initial bulk modulus $K_{0}$, and Poisson’s ratio ν) as given in Equation (2).

(2) $$\mu_{0}=\sum_{i=1}^{2}\mu_{i},\;K_{0}=\overset{2}{\underset{i=1}{\sum }}2\mu_{i}\left(\frac{1}{3}+\beta_{i}\right),\;v_{i}=\frac{\beta_{i}}{1+2\beta_{i}}$$

In a uniaxial compression test, the material is compressed along one axis but is allowed to deform freely in the transverse directions. Denoting the principal stretch in the loading direction as $\lambda_{L}$ and assuming isotropic deformation in the unloaded plane ($\lambda_{2}=\lambda_{3}$), Equation (1) can be reduced to

(3) $$U\left(\lambda_{1},\lambda_{2}\right)=\overset{2}{\underset{i=1}{\sum }}\frac{2\mu_{i}}{\alpha_{i}^{2}}\left\{\lambda_{L}^{\alpha_{i}}+2\lambda_{2}^{\alpha_{i}}-3+\frac{1}{\beta_{i}}\left[{\left(\lambda_{L}{\lambda_{2}}^{2}\right)}^{-\alpha_{i}\beta_{i}}-1\right]\right\}$$

To obtain the nominal stress σ in the loading direction, we take the partial derivative of the strain energy density W with respect to the principal stretch $\lambda_{L}$, i.e., $\sigma =\partial W/\partial \lambda_{L}$. Then, substituting the relationship between stretch and engineering strain (ε=λ−1) into the resulting expression yields the stress as a function of strain:

(4) $$\sigma_{L}=\frac{2}{1+\varepsilon_{L}}\overset{2}{\underset{i=1}{\sum }}\frac{\mu_{i}}{\alpha_{i}}\left\{{\left(1+\varepsilon_{L}\right)}^{\alpha_{i}}-{\left[\left(1+\varepsilon_{L}\right){\left(1-v_{i}\varepsilon_{L}\right)}^{2}\right]}^{-\alpha_{i}\beta_{i}}\right\}$$

where $\sigma_{L}$ is the nominal compressive stress (MPa), and $\varepsilon_{L}$ is the engineering strain (dimensionless).

The silicone rubber foam used in this study has a porosity of 55%. Referring to existing research, its porous structure provides very weak lateral constraint, and the lateral strain during compression can be neglected. Following the practice in the literature [14,16,20], Poisson’s ratio is set to zero in the model (ν=0). Under this assumption, parameter $\beta_{i}\approx 0$, which greatly simplifies the constitutive relation. Substituting $\beta_{i}\approx 0$ into Equation (4) and noting that the volumetric deformation term vanishes, we obtain the simplified form:

(5) $$\sigma_{L}=\frac{2}{1+\varepsilon_{L}}\overset{2}{\underset{i=1}{\sum }}\frac{\mu_{i}}{\alpha_{i}}[{\left(1+\varepsilon_{L}\right)}^{\alpha_{i}}-1]$$

The excellent fitting results presented in Section 4.1 confirm the validity of this simplification.

To analyze the evolution of the model parameter with aging time, we examine the relationship between the experimentally measurable aging indicators (compression set and stress relaxation) and model parameter as follows.

**Figure 1:**
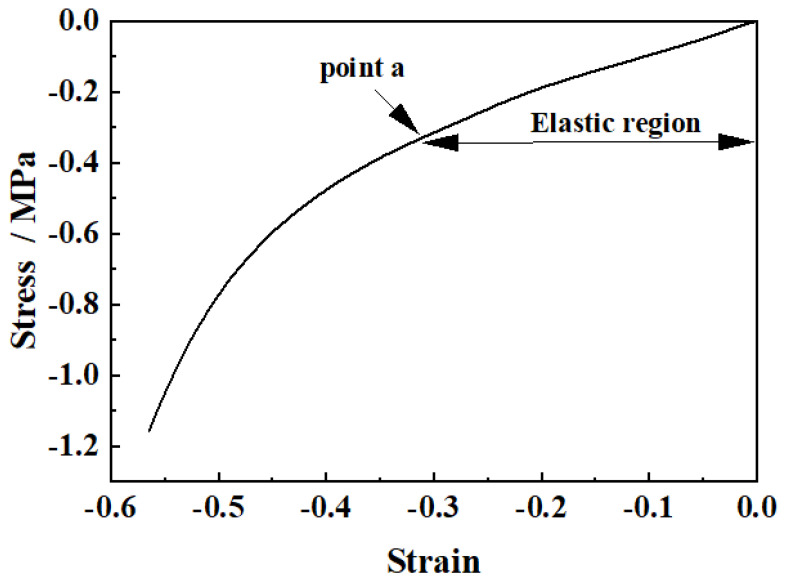
Typical stress–strain curves of silicon foam.

As shown in Figure 1, the stress–strain curve is approximately linear in the small-strain region, and its slope is taken as the initial elastic modulus E, i.e., the stress-to-strain ratio at point a, given by Equation (6).

(6) $$E_{0}=\frac{\sigma_{a}}{\varepsilon_{a}}$$

After aging, the stress and strain at the same relative position (point *a*) become $\sigma_{a}^{t}$, and the strain becomes $\varepsilon_{a}^{t}$. In the following, we derive how $\sigma_{a}$ and $\varepsilon_{a}$ change with aging and how they relate to the experimentally measurable aging indicators, i.e., compression set *C* and stress relaxation *L*.

Assuming that the initial height of the silicone foam material is $h_{0}$, the recovery height after aging is $h_{t}$, and the height of point *a* is $h_{a}$, then the strain at point *a* after aging can then be given by Equation (7).

(7) $$\varepsilon_{a}^{t}=\frac{h_{a}-h_{t}}{h_{t}}$$

Referring to GB/T 7759 [21], the material compression set $C_{t}$ is defined as

(8) $$C_{t}=\frac{h_{0}-h_{t}}{h_{0}-h_{s}}$$

where $h_{s}$ is the height of the limiter. From Equation (8), the recovered height after aging can be expressed as

(9) $$h_{t}=h_{0}+\left(h_{s}-h_{0}\right)C_{t}$$

Substituting Equation (9) into Equation (7), the strain $\varepsilon_{a}^{t}$ after aging can be expressed as

(10) $$\varepsilon_{a}^{t}=\frac{h_{a}-[h_{0}+\left(h_{s}-h_{0}\right)C_{t}]}{h_{0}+\left(h_{s}-h_{0}\right)C_{t}}$$

The stress retention coefficient $L_{t}$ is defined as the ratio of post-aging stress to the initial stress at the same strain level:

(11) $$L_{t}=\frac{\sigma_{t}}{\sigma_{0}}$$

where $\sigma_{0}$ is the stress before aging, and $\sigma_{t}$ is the stress after aging.

Thus, the post-aging stress at point a is

(12) $$\sigma_{a}^{t}=\sigma_{a}L_{t}$$

By introducing the post-aging stress $\sigma_{a}^{t}$ and strain $\varepsilon_{a}^{t}$ into Equation (6), the relationship between the initial elastic modulus *E* and compression set *C* and stress relaxation *L* of silicon foam is obtained.

(13) $$E_{t}=\frac{\sigma_{a}^{t}}{\varepsilon_{a}^{t}}=\frac{\sigma_{a}L_{t}[h_{0}+\left(h_{s}-h_{0}\right)C_{t}]}{h_{a}-[h_{0}+\left(h_{s}-h_{0}\right)C_{t}]}$$

Given $E_{0}=2\mu_{0}=2\left(\mu_{1}+\mu_{2}\right)$, the relationship between the parameter $\mu_{i}$ and compression set $C_{t}$ and stress relaxation $\sigma_{L}$ is obtained.

## 3. Aging Test

### 3.1. Materials

The material used in this aging test is a kind of elevated temperature silicone rubber foam, which was purchased from Xueru seal Co., Ltd., Hangzhou, Zhejiang, China. It had a white, adhesive layer with a cell density of 55%. For the aging test, the foam was cut into cube samples with dimensions of 10 mm × 10 mm × 10 mm.

### 3.2. Test Conditions and Procedures

**Figure 2:**
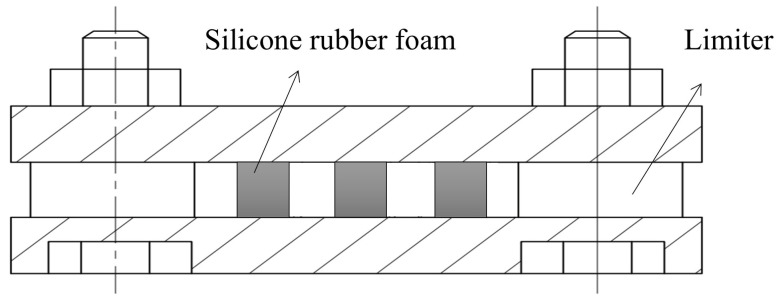
Schematic of fixture.

The cube-shaped samples were mounted in a fixture (according to GB/T 1683 [22]), where a constant aging compressive strain of 40% was maintained using spacers, as shown in Figure 2. After being held for 1 day, the samples were removed from the pre-compression device. Following a 24 h stabilization period at room temperature, their height, load, and stress–strain curves were measured to establish the unaged baseline data.

Subsequently, the samples were placed back into the fixture and subjected to accelerated thermal aging in an air-circulating oven (Shanghai Testing Instrument Factory Co., Ltd., Shanghai, China). According to Jiang et al. [23], the oxidative aging mechanism of silicone rubber remains unchanged at temperatures up to 152 °C. Although a lower temperature better simulates real-world aging, an excessively long test duration would be required. To balance experimental efficiency and aging representativeness, 85 °C was selected as the aging temperature, with a total aging duration of up to 768 h. Moreover, the aging process was faster in the early stage and slower in the later stage. Therefore, samples were removed at specific intervals (48, 120, 192, 288, 384, 480, 576, and 768 h) for testing. The compression set was measured concurrently using a thickness gauge with an accuracy of 0.01 mm. Stress relaxation and stress–strain curves were obtained using a universal testing machine at a loading rate of 10.0 mm/min.

**Figure 3:**
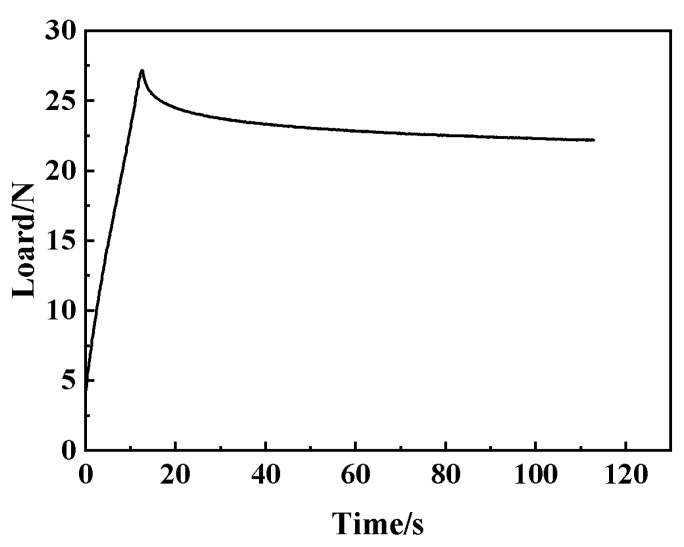
Stress relaxation curve of silicone foam.

To establish the relationship between the initial elastic modulus ($E_{0}$), compression set, and stress relaxation within the elastic region, stress relaxation was measured under a 30% strain using a discontinuous stress relaxation test method (the compression set data were also converted to correspond to the 30% strain condition). The resulting stress relaxation curve for the silicone foam is presented in Figure 3. The curve indicates a significant initial change in the load applied to the foam, which gradually stabilizes between 600 and 1000 s. Therefore, the load at 1000 s was taken as the stabilized load value. To ensure the reliability and repeatability of the mechanical test results, three parallel samples were prepared for each aging condition. The compression set and stress relaxation results were averaged, while the stress–strain curves exhibited good repeatability, and one representative curve was selected for constitutive model fitting.

## 4. Results and Discussion

### 4.1. Parameter Identification

**Figure 4:**
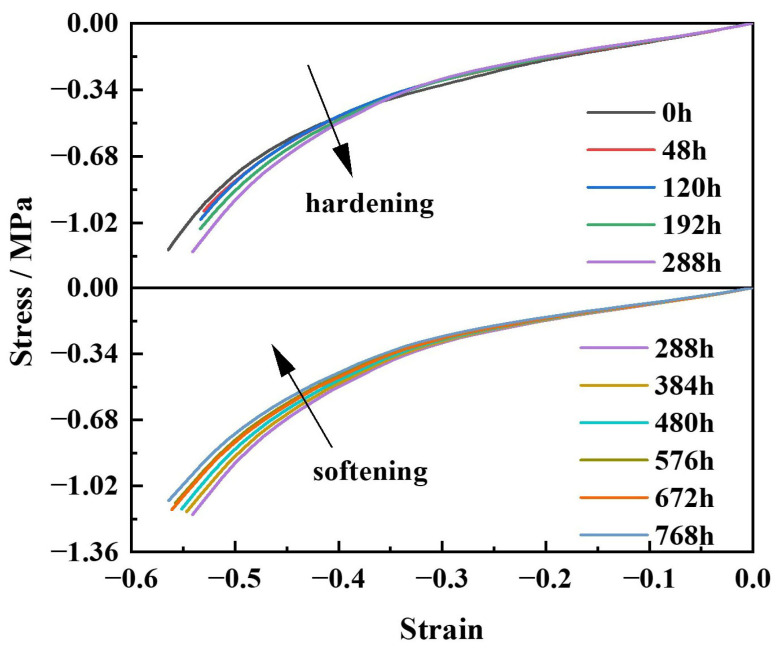
Stress–strain curves of silicone rubber foam aged at different times.

**Figure 5:**
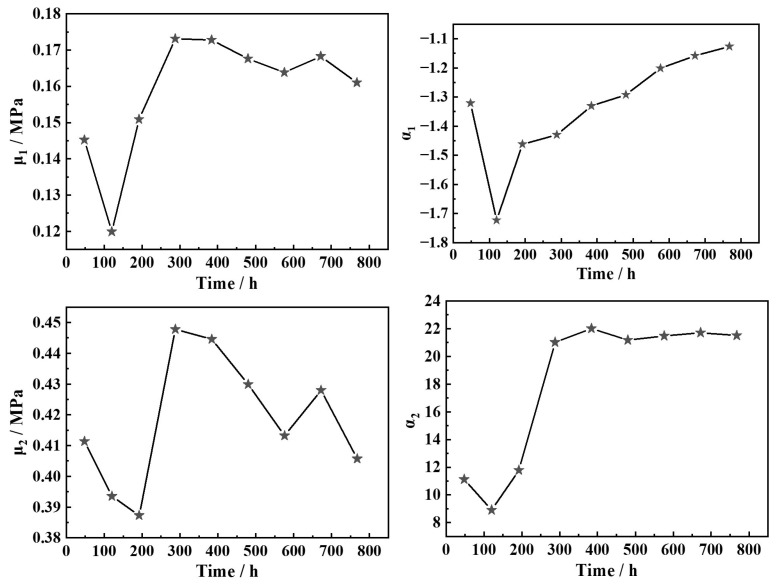
Evolution of constitutive model parameters with aging time: $\mu_{1}$, $\alpha_{1}$, $\mu_{2}$, and $\alpha_{2}$. The star symbols represent the model parameters fitted at each aging time.

The evolution of the stress–strain curve of the silicone rubber foam with increasing aging time is shown in Figure 4. The stress–strain response shows a non-monotonic trend during aging: the material initially stiffens and then gradually softens. The apparent fluctuations in Figure 4 reflect real mechanical responses driven by the competition between crosslinking and chain scission during aging [24,25]. From 48 to 288 h, crosslinking dominates and the material stiffens; from 288 to 768 h, chain scission prevails and the material softens. To further analyze this complex mechanical evolution, a second-order Ogden model was established through the nonlinear least squares fitting of uniaxial compression stress–strain data, where the fitting objective was to minimize the sum of squared residuals between experimental and predicted stresses. The temporal variations in the model parameters μ_1_, α_1_, μ_2_, and α_2_ are summarized in Table 1. As shown in Figure 5, the parameters themselves exhibit non-monotonic variation over time, which further complicates the development of a robust aging constitutive model. The fluctuations in Figure 5 arise from two aspects: the competing crosslinking/chain scission reactions during aging and severe parameter coupling with multiple local optima in four-parameter fitting, which causes unstable parameter values. Taking the 768 h aged sample as an example, repeated fitting of the same stress–strain curve yields three distinct parameter combinations (see Table 1), all achieving the same high coefficient of determination (R^2^ = 0.9999). This indicates that different parameter sets can fit the experimental data equally well but lack consistent physical interpretability, which is the core manifestation of the local optimum problem.

**Figure 6:**
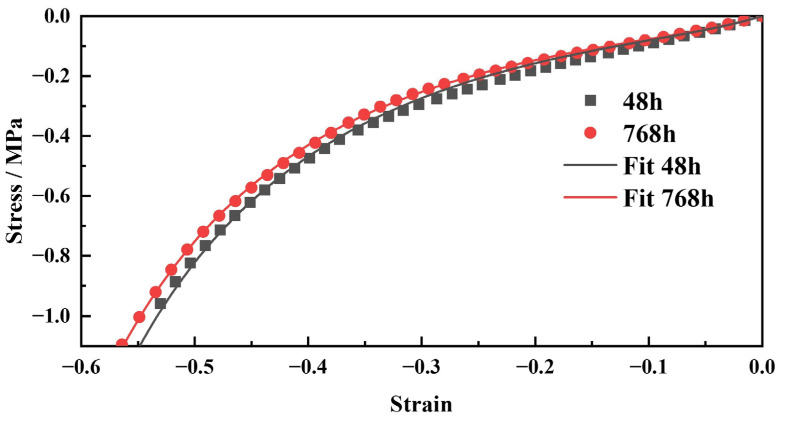
Constitutive model fitting results for materials aged for 48 h (gray squares and line) and 768 h (red circles and line).

To eliminate the influence of local optima and effectively investigate parameter evolution during aging, we fix three parameters and allow only μ_1_ to vary with time. Studies have shown that, for the same material, fixing certain parameters has a negligible impact on the accuracy of the constitutive model [15,16]. For the second-order Ogden model, μ_1_ dominates the initial shear modulus and directly reflects the initial stiffness of the crosslinked network of silicone rubber foam, which governs the aging-induced stiffening (crosslinking) and subsequent softening (chain scission). In contrast, μ_2_ describes hardening at large strains and is barely affected by aging; α_1_ and α_2_ characterize chain limit deformation and remain nearly constant during aging. Therefore, in this work, μ_1_ is designated as the time-varying parameter, while α_1_ = −1.2, α_2_ = 21, and μ_2_ = 0.4 are held constant. By fixing α_1_, α_2_, and μ_2_, the coupling is completely removed, leaving only μ_1_ to be optimized. This guarantees a unique solution and effectively avoids local optima. Under these conditions, the values of the model parameter μ_1_ are listed in Table 2. Figure 6 presents the fitting results for the silicone rubber foam aged for 48 h (representing the worst fit) and for 768 h (the longest aging duration). Based on the coefficient of determination (R^2^) and the fitted curves, the model accurately captures the stress–strain behavior at all stages of the aging process, confirming the validity of our assumptions.

Our approach significantly reduces the number of parameters with only a minor compromise in fitting accuracy. Consequently, the evolution of the stress–strain curve can be predicted through parameter μ_1_. Specifically, an increase in μ_1_ corresponds to the hardening stage of the curve (aging from 48 to 288 h), while a decrease in μ_1_ corresponds to the softening stage (aging from 288 to 768 h). Based on these findings, the modified constitutive model is formulated as follows:

(14) $$\sigma_{L}=\frac{2}{1+\varepsilon_{L}}\times \left\{\left\{\frac{\mu_{1}(t)}{-1.2}\left[{\left(1+\varepsilon_{L}\right)}^{-1.2}-1\right]+\frac{0.4}{21}\left[{\left(1+\varepsilon_{L}\right)}^{21}-1\right]\right.\right\}$$

where $\mu_{1}(t)$ is a time-dependent parameter.

According to $E_{0}=2\left(\mu_{1}+\mu_{2}\right)$ and Equation (14), the equations of parameter *μ*_1_ with compressive set and stress relaxation are obtained.

### 4.2. Establishment of Aging Constitutive Model

#### 4.2.1. Degradation Trajectory of Compression Set and Stress Relaxation

**Figure 7:**
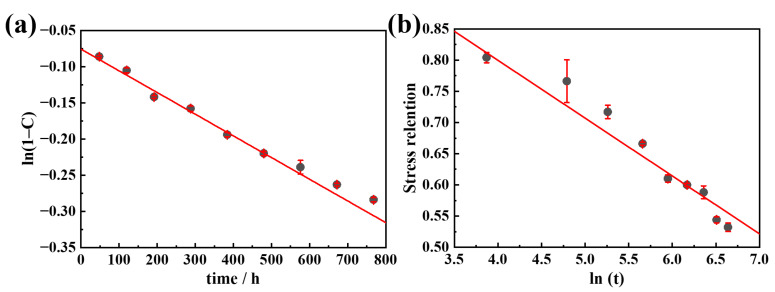
Fitting curves of (**a**) compression set and (**b**) stress relaxation for silicone rubber foam after different aging times. In both panels, the discrete dots correspond to the experimentally measured data, the red solid lines represent the fitting results obtained from Equation (15) (for compression set) and Equation (16) (for stress relaxation), respectively, and the error bars show the standard deviation of three parallel measurements.

Previous studies have shown that during the aging process of silicone rubber foam, its compression set typically increases with aging time following an exponential function, reflecting the accumulation of irreversible deformation in the material. In contrast, stress relaxation behavior often adheres to a logarithmic relationship, characterizing the decay of stress over time under constant strain. Based on this, the present study established mathematical expressions for the evolution of compression set and stress relaxation with aging time by fitting experimental data, namely Equations (15) and (16). The fitting results are shown in Figure 7, which presents both the experimental data points and the fitted curves, demonstrating good agreement between them. Consequently, the degradation trajectories of compression set and stress relaxation during thermal aging can be determined, providing a basis for establishing the time-dependent evolution of parameters in subsequent constitutive models.

(15) $$\ln(1-C_{t})=-0.0003t-0.0757$$

(16) $$L_{t}=1.1705-0.0927\ln(t)$$

where t denotes aging time, *Cₜ* denotes compression set, and *Lₜ* denotes stress relaxation.

#### 4.2.2. Model Validation

Given the non-monotonic variation in model parameters over time, this study established the evolution of model parameter μ_1_ with aging time by analyzing the relationship among compression set, stress relaxation, and elastic modulus (Equation (13)), based on the fundamental relationship between elastic modulus and model parameters (*E*_0_ = 2(μ_1_ + μ_2_)).

In applying Equation (13), stress relaxation data were measured using a universal testing machine with an intermittent loading method, with a compression limit height set to 7 mm. The initial contact position of the testing machine was determined visually by adjusting the pressure sensor to contact the foam surface, which introduced a limit height error Δ*h*. Therefore, in the calculations, the actual initial height *ha* was corrected to 7 + Δ*h*. In the compression set test, the limit height was set to 6 mm, and the actual recovered height of the specimen after stress release was 9.35 mm. By substituting *h*_0_ = 9.35 mm and *hs* = 6 mm into Equation (13), the corresponding calculations were performed.

(17) $$E_{t}=-\frac{\sigma_{0}L_{t}[9.35-3.35C_{t}]}{2.35-3.35C_{t}-\Delta h}$$

where $\sigma_{0}$ is the initial stress before aging at a specified strain.

To identify the parameters $\sigma_{0}$ and Δh, Equation (13) is transformed as follows:

(18) $$L_{t}/E_{t}=-\frac{2.35-3.35C_{t}-\Delta h}{\sigma_{0}\left(9.35-3.35C_{t}\right)}$$

The data for compression set, stress relaxation, and elastic modulus after aging for different times are summarized in Table 3.

**Figure 8:**
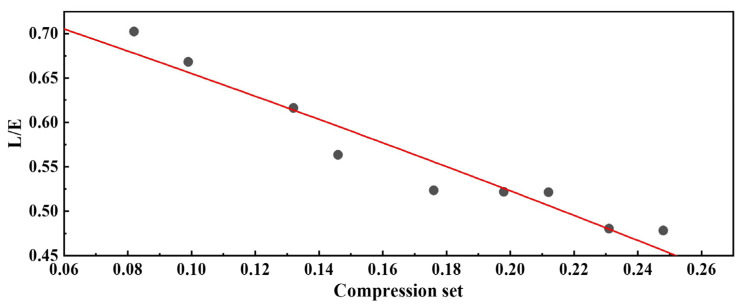
The fitting results of compression set (*C*) vs. the ratio of stress retention factor to initial elastic modulus (*L/E*). The scatter points represent experimental data, and the solid line is the fitting curve derived from Equation (19).

Substituting the compression set, *C*, and the ratio of the stress retention coefficient to the elastic modulus, *L/E*, into Equation (18) for curve fitting yields the results shown in Figure 8. The fitting results show that $\sigma_{0}=-0.245\;\mathrm{MPa}$ and Δh=0.568 mm, which agree with the experimental results (e.g., σ_0_, exp = −0.24 MPa), confirming the validity of Equation (13), which relates the initial elastic modulus to the compression set and stress relaxation. This agreement provides a novel approach for constructing aging constitutive models. Furthermore, the following relation can be derived from Equation (18):

(19) $$\frac{1}{E_{t}}=-\frac{1}{L_{t}\sigma_{0}}\left(1-\frac{7+\Delta h}{9.35-3.35C_{t}}\right)$$

#### 4.2.3. Aging Constitutive Model

Based on Equation (13) and in conjunction with the degradation trajectories of compression set and stress relaxation, the evolution of the elastic modulus with aging time can be described as follows:

(20) $$\frac{1}{E_{t}}=\frac{1}{a+b\ln t}\left(1+\frac{1}{c*\exp(d*t)}\right)$$

where *a*, *b*, *c*, and *d* are constants.

**Figure 9:**
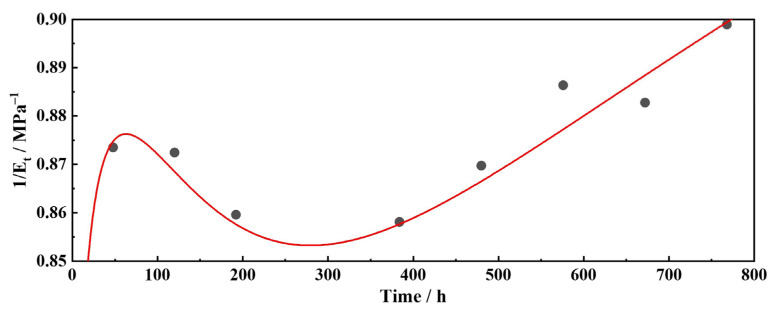
Fitting results of inverse of initial elastic modulus of silicone rubber foam with aging time. Symbols denote experimental data, and the line is the fit from Equation (20).

Parameter fitting was conducted for this equation, and the results are presented in Figure 9, with the following fitted parameter values: *a* = 1.1886, *b* = −0.1164, *c* = 2.785, and *d* = 0.007012.

**Figure 10:**
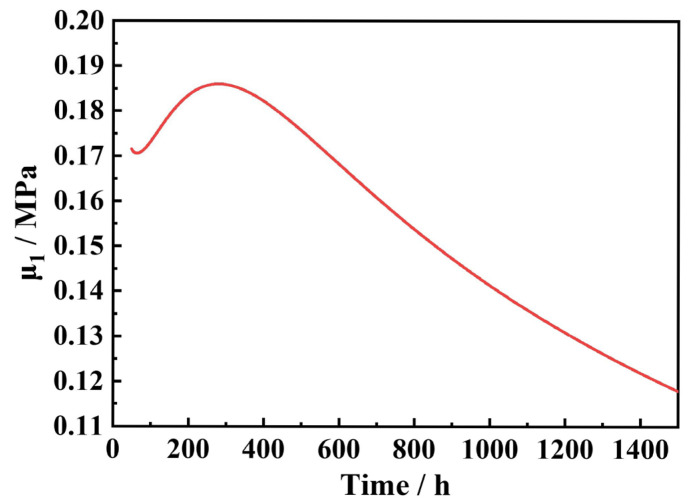
Prediction results of aging model parameters *μ*_1_ at different times.

First, based on the constitutive relation E_0_ = 2(μ_1_ + μ_2_) and the assigned parameter μ_2_ = 0.4, Equation (20) is substituted to derive the explicit expression for the temporal evolution of the model parameter μ_1_, which is given by Equation (21) and whose predicted trend is illustrated in Figure 10. Finally, by integrating the model framework of Equation (14) with the parameter evolution law from Equation (21), the aging constitutive model for the silicone rubber foam is constructed.

(21) $$\mu_{1}(t)=\frac{1}{2}\frac{\left(1.1886-0.1164\ln(t)\right)\times 2.785\exp(0.007012t)}{1+2.785\exp(0.007012t)}-0.4$$

## 5. Conclusions

This study proposes and validates a novel constitutive model for describing the thermomechanical aging behavior of silicone rubber foam through systematic experimental characterization and theoretical modeling. To address the limitations of traditional multi-parameter models, such as their susceptibility to local optima and lack of clear physical interpretability of parameters, this work innovatively adopts a parameter simplification strategy. Based on the second-order Ogden model framework, three parameters are fixed (α_1_ = −1.2, α_2_ = 21, μ_2_ = 0.4), with only μ_1_ designated as the time-varying parameter. This approach reduces the number of parameters to be fitted from four to one, significantly lowering the complexity and risk of multiple solutions in model identification, while maintaining excellent fitting accuracy (R^2^ ≥ 0.9966) throughout the entire aging cycle.

Accelerated thermal aging experiments were conducted to obtain data on stress–strain curves, compression set, and stress relaxation during the aging of silicone rubber foam. Analysis reveals that the material’s stress–strain response exhibits a non-monotonic trend, characterized by initial hardening followed by gradual softening. Based on the fundamental relationship between elastic modulus and model parameters (E_0_ = 2(μ_1_ + μ_2_)), and in conjunction with the degradation trajectory formulas for compression set and stress relaxation, an explicit function describing the temporal evolution of parameter μ_1_ (Equation (21)) was ultimately derived. Substituting this function into the simplified Ogden model (Equation (14)) yields an aging constitutive model containing only a single time parameter, with parameters that possess clear physical significance. This model provides a more efficient and reliable theoretical tool and a novel modeling approach for the rapid assessment of aging performance, lifetime prediction, and finite element simulation of structural components made from silicone rubber foam.

## Figures and Tables

**Table 1:** Constitutive model parameters of silicone rubber foam aged at different times.

Aging Time	$\mu_{1}$	$\alpha_{1}$	$\mu_{2}$	$\alpha_{2}$	R-Square
48 h	0.1452	−1.321	0.4114	11.12	0.9999
120 h	0.1199	−1.723	0.3935	8.8983	0.9999
192 h	0.1509	−1.462	0.3873	11.78	0.9999
288 h	0.1731	−1.43	0.4478	21.02	0.9999
384 h	0.1728	−1.331	0.4446	22.02	0.9999
480 h	0.1676	−1.292	0.4299	21.18	0.9999
576 h	0.1638	−1.201	0.4132	21.48	0.9999
672 h	0.1683	−1.158	0.428	21.71	0.9999
768 h	0.1610	−1.126	0.4057	21.51	0.9999
	0.1682	−1.160	0.4241	21.42	0.9999
	0.1686	−1.156	0.4297	21.84	0.9999

**Table 2:** The variation law of model parameter *μ*_1_ with time, when *α*_1_ = −1.2, *μ*_2_ = 0.4, and *α*_2_ = 21.

Aging Time	*μ*_1_	R-Square
48	0.1724	0.9966
120	0.1731	0.9981
192	0.1817	0.9992
288	0.1910	0.9996
384	0.1827	0.9998
480	0.1749	0.9998
576	0.1641	0.9999
672	0.1664	0.9998
768	0.1562	0.9998

**Table 3:** Compression set, stress relaxation, and elastic modulus after aging at different times at 85 °C.

Aging Time/h	Compression Set *C*	Stress Relaxation *L*	Elastic Modulus *E*	*L/E*
48	0.082	0.804	1.1448	0.7023
120	0.099	0.766	1.1462	0.6683
192	0.132	0.717	1.1634	0.6163
288	0.146	0.666	1.1820	0.5635
384	0.176	0.610	1.1654	0.5234
480	0.198	0.600	1.1498	0.5218
576	0.212	0.588	1.1282	0.5212
672	0.231	0.544	1.1328	0.4802
768	0.248	0.532	1.1124	0.4782

## Data Availability

The original contributions presented in this study are included in the article. Further inquiries can be directed to the corresponding authors.
